# Exploring the research landscape of COVID-19-induced olfactory dysfunction: A bibliometric study

**DOI:** 10.3389/fnins.2023.1164901

**Published:** 2023-03-24

**Authors:** Zhirong Yang, Yukun Ma, Wei Bi, Jingqian Tang

**Affiliations:** ^1^Library of Zhuhai Campus, Jinan University, Zhuhai, China; ^2^Department of Otorhinolaryngology-Head and Neck Surgery, The First Affiliated Hospital of Jinan University, Guangzhou, China; ^3^Department of Neurology, The First Affiliated Hospital of Jinan University, Guangzhou, China; ^4^Clinical Neuroscience Institute, The First Affiliated Hospital of Jinan University, Guangzhou, China; ^5^Department of Subject Service and Consultation, Jinan University Library, Guangzhou, China; ^6^Intellectual Property Information Service Center, Jinan University, Guangzhou, China

**Keywords:** COVID-19 pandemic, long COVID, olfactory dysfunction, co-citation analysis, co-word analysis, cluster analysis

## Abstract

Since the outbreak of COVID-19, olfactory dysfunction (OD) has become an important and persistent legacy problem that seriously affects the quality of life. The purpose of this paper is to quantitatively analyze and visualize the current research status and development trend of COVID-19 related OD by using VOSviewer software. Based on the Web of Science database, a total of 1,592 relevant documents were retrieved in January 2023, with publication time spanning from 2020 to 2023. The bibliometric analysis revealed that the most influential research results in the field of COVID-19 related OD were concentrated in journals of related disciplines such as otorhinolaryngology, medicine, general and internal, virology, neurosciences, etc. The knowledge base of the research is mainly formed in two fields: COVID-19 clinical research and OD specialized research. The research hotspots are mainly concentrated in six directions: COVID-19, long COVID, smell, anosmia, OD, and recovery. Based on the results of the bibliometric analysis, the temporal trends of COVID-19 related OD studies were visually revealed, and relevant suggestions for future research were proposed.

## 1. Introduction

The novel coronavirus disease 2019 (COVID-19), caused by the severe acute respiratory syndrome coronavirus-2 (SARS-CoV-2), was first reported in late 2019 and quickly spread globally, leading to the declaration of a pandemic by the World Health Organization on March 11, 2020 (Sharma et al., 2020). A growing body of evidence suggests that the most common symptom of COVID-19 infection is the loss or diminished sense of smell (hyposmia) (Gori et al., 2020; Dawson et al., 2021; de Melo et al., 2021; Gupta et al., 2021). Before the COVID-19 pandemic, other factors such as viral infections, sinus disease, and head trauma could also cause olfactory loss (Desai and Oppenheimer, 2021). However, SARS-CoV-2 has been found to cause a more severe form of hyposmia compared to other seasonal cold viruses (Haehner et al., 2022). OD is also one of the most common neurological complications reported among patients with COVID-19 (Azizi and Azizi, 2020; Wei et al., 2022).

OD associated with COVID-19 has a significant impact on quality of life and may lead to several negative outcomes, such as malnutrition, weight loss, food poisoning, and exposure to hazardous chemicals (Gómez-Iglesias et al., 2020; Glezer et al., 2021). Moreover, individuals with COVID-19 who experience olfactory loss are more likely to suffer from poor sleep quality, high levels of fatigue, and depression compared to those who do not (Alqahtani et al., 2022).

In response to the COVID-19 pandemic, many researchers have focused on studying OD. Several literature reviews, systematic reviews, and meta-analyses have been conducted, including a meta-analysis of data from 24 studies of 8,438 patients with COVID-19 from 13 countries (Agyeman et al., 2020) and a meta-analysis of 11,074 patients with confirmed COVID-19 in 51 studies (Aziz et al., 2021). These studies have shown that OD is a common and important extrapulmonary manifestation of COVID-19.

However, despite these efforts, there have been only a limited number of comprehensive, quantitative analyses, and visualizations of COVID-19 related OD using bibliometrics. The data from these analyses were collected before 2021 (Hu et al., 2022; Zyoud et al., 2022), making it challenging to identify the most recent research directions in this field. To address this gap, this study will perform a comprehensive review and visualization of existing COVID-19 related OD research using a bibliometric approach. Specifically, co-citation analysis, co-word analysis, and cluster analysis will be applied to data retrieved from the Web of Science using VOSviewer. The results of this analysis will identify the knowledge base and research hotspots of COVID-19 related OD, reveal the temporal trends of this research, and provide recommendations for future COVID-19 related OD research.

## 2. Data source and analysis method

### 2.1. Data source

To ensure the authority and comprehensiveness of the study data, the Web of Science Core Collection was used as the data source, from which the Science Citation Index Expanded (SCI-EXPANDED) and Social Sciences Citation Index (SSCI) databases. The search terms for the COVID-19 Pandemic included “COVID-19,” “Corona Virus,” “Coronavirus,” and “2019-nCoV.” The search terms for Olfaction research included “olfactory,” “olfaction,” and “smell.” The search query was set as “TS = (COVID-19 or Corona Virus or Coronavirus or 2019-nCoV) AND TS = (olfactory or olfaction or smell).” The types of literature were limited to articles, review articles, and early access, with a publication year restricted to 2020 to present. The search was conducted on January 23, 2023, and a total of 1,592 relevant literature was finally retrieved and used as the data source for this study.

Table 1 presents the number of papers investigating OD in the context of the COVID-19 pandemic. As of January 23, 2023, 391 papers were published in 2020, with 655 papers in 2021, 541 papers in 2022, and 5 papers having been published in 2023. In the 1,592 relevant literature, there were 1,222 articles (76.76%), 359 review articles (22.55%), and 11 papers in early access type (0.69%). This indicates a significant amount of in-depth studies on COVID-19 related OD have been conducted since 2020, and suggests the rapidly evolving nature of the COVID-19 pandemic has necessitated a large number of Review Articles to keep up with the latest literature data.

**Table 1:** The status of literature publications (2020–2023).

**Publication year**	**Articles**	**Review articles**	**Count**
2020	278	113	391
2021	510	144	654
2022	430	101	51
2023	4	1	5
Total	1,222 (76.76%)	359 (22.55%)	1,581 (99.31%)

According to Table 2, the top 10 countries or regions involved in COVID-19 related research are highly concentrated. Out of the 104 countries or regions worldwide, the United States leads with a total of 395 documents, representing 24.812% of the scientific output. This is followed by Italy with 216 articles, accounting for 13.568%. Other countries such as England, Germany, France, and others have also made significant contributions to the research output. The top 10 countries alone make up 93.72% of the total publications on the subject, highlighting a noticeable concentration trend.

**Table 2:** The geographical distribution (Top 10).

**Country/region**	**Count**	**Percentage**	**Country/region**	**Count**	**Percentage**
USA	395	24.812	Turkey	108	6.784
Italy	216	13.568	Peoples R China	99	6.219
England	164	10.302	Spain	88	5.528
Germany	138	8.668	India	79	4.962
France	133	8.354	Belgium	72	4.523

### 2.2. Analysis method

**Figure 1:**
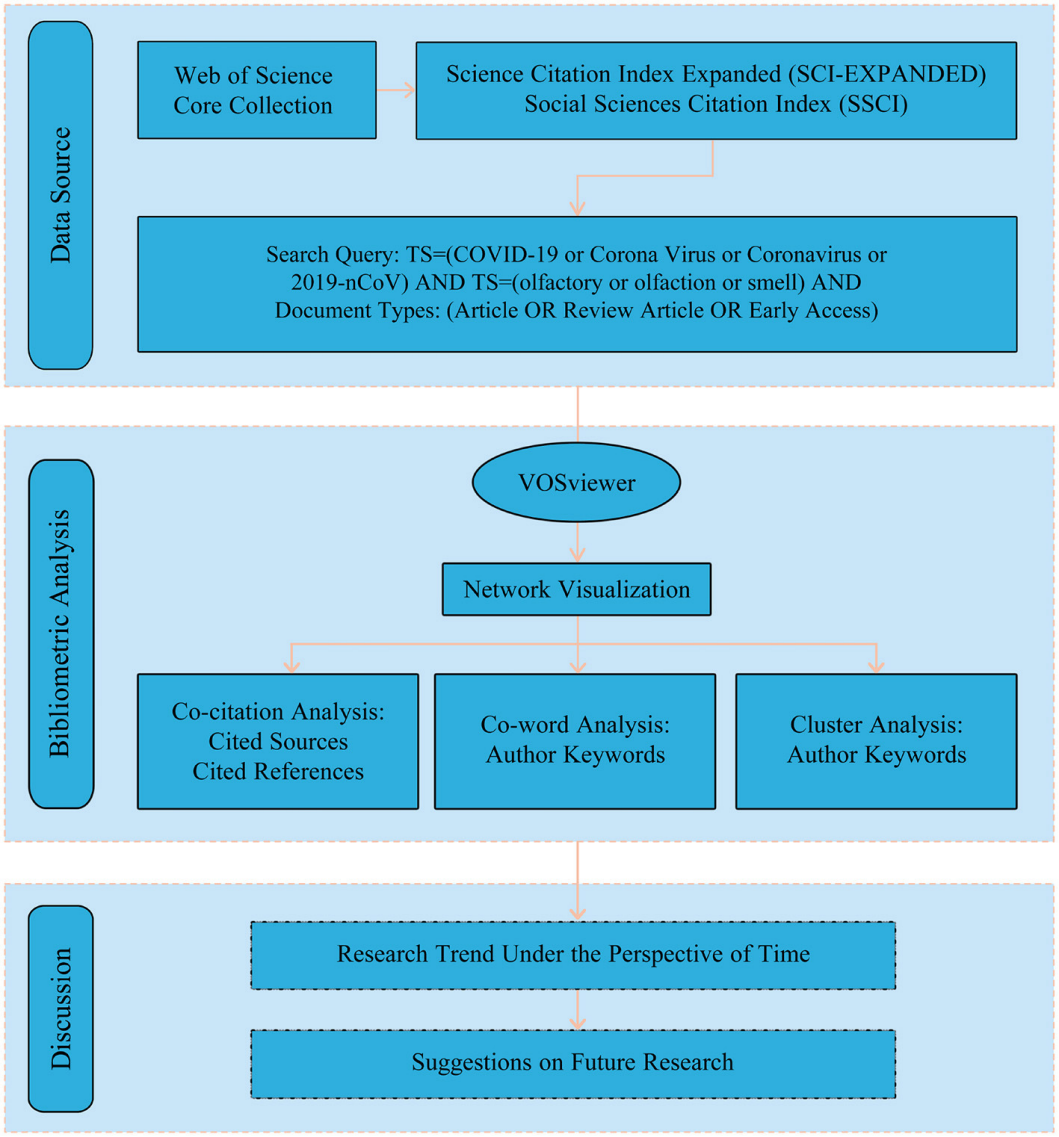
The structure of this review.

Since 1969 (Pritchard, 1969), bibliometric analysis has been widely used in scientific and application fields (Ellegaard and Wallin, 2015; Huang et al., 2016; Xu et al., 2021). Since 2020, bibliometric analysis has been used (Zhang et al., 2020; Lin et al., 2022; Pang et al., 2022; Xie et al., 2022) to help researchers grasp the knowledge base, hot spots, and trends in the research field of COVID-19. VOSviewer, a powerful bibliometric analysis software developed by van Eck and Waltman in 2010, which offers advanced graphical representation capabilities for mapping knowledge units and their relationships within the research literature (van Eck and Waltman, 2010). In order to perform bibliometric analysis of the knowledge base and research hotspots of COVID-19 related OD research, VOSviewer was used as the knowledge mapping analysis tool in this paper, and the software version was VOSviewer_1.6.19. First, the full record and cited references data of the retrieved 1,592 literature references data retrieved were imported into VOSviewer, and then co-citation analysis, co-word analysis and cluster analysis were performed. The research structure of this review is shown in Figure 1.

## 3. Results

### 3.1. Most co-cited journals

**Figure 2:**
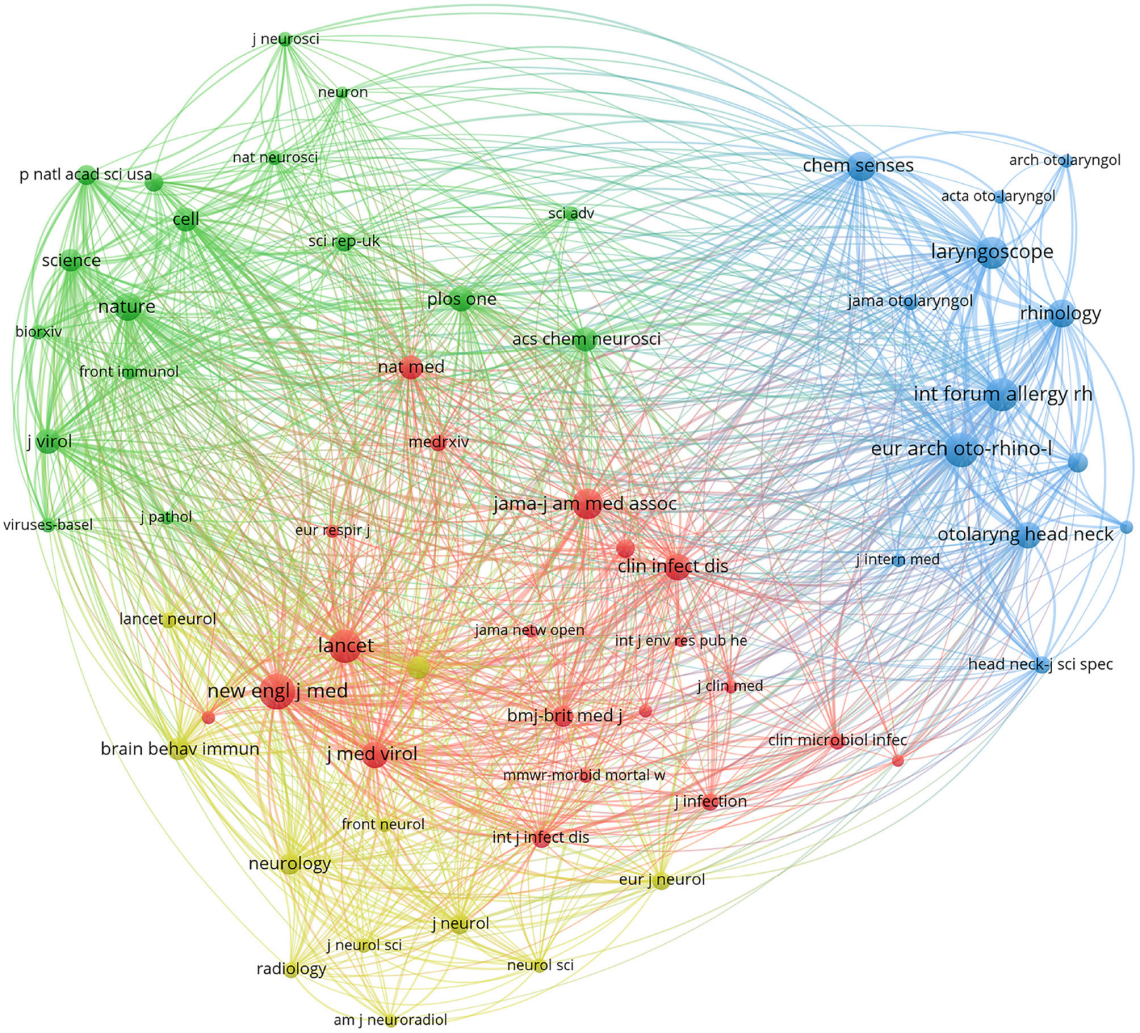
The mapping of cited sources co-citation analysis.

As depicted in Figure 2, the results of the co-citation analysis of cited sources are presented in a mapping format, produced using the VOSviewer software. In order to ensure the relevance of the results, the authors chose to only include cited sources with a citation frequency exceeding 200. Out of the 91 cited sources present in the data set, 61 sources met this criterion.

The visual representation in Figure 2 utilizes nodes to symbolize sources and links to indicate the co-citation relationships between them. Sources that share close co-citation relationships are marked with the same color. The results of the analysis highlight that the majority of the co-cited sources in COVID-19 related ocular disease research are journal articles. Notable journals such as the *New England Journal of Medicine, European Archives of Oto-Rhino-Laryngology, Lancet*, and *Nature* occupy a central position and are closely distributed, showcasing their significant influence on the topic of COVID-19 related ocular disease research and their interrelatedness.

Table 3 showcases the top ten journals as determined by the Total Link Strength (TLS) metric in VOSviewer. The *New England Journal of Medicine, European Archives of Oto-Rhino-Laryngology*, and *International Forum of Allergy & Rhinology* stand out as the journals with higher Local Citation Score (LCS) and TLS values compared to other journals. This implies that these journals possess a strong reputation in the field of COVID-19 related ocular disease research. Eight of the top ten journals are published in the United States while the remaining two are published in Europe, indicating a concentration of COVID-19 related ocular disease research in these regions. The analysis results in Table 3 also reveal that the most impactful journals for COVID-19 related ocular disease research primarily belong to the categories of otorhinolaryngology, medicine, general and internal medicine, virology, and neurosciences.

**Table 3:** Highly total link strength cited journals (Top 10).

**Journal**	**JCR category**	**Country of publisher**	**LCS**	**TLS**
New England journal of medicine	GIM	USA	1,611	45,004
European archives of Oto-Rhino-Laryngology	ORL	USA	1,418	39,503
International forum of allergy rhinology	ORL	USA	1,335	37,517
Lancet	GIM	USA	1,326	35,443
Laryngoscope	MRE/ORL	USA	1,250	34,712
Nature	MS	Germany	1,001	34,552
Chemical senses	BS/FST/NEU/PHYS	England	1,099	31,394
Journal of medical virology	VIR	USA	901	29,250
Cell	BMB/CB	USA	714	29,085
Journal of virology	VIR	USA	822	28,746

GIM, Medicine, General, and Internal; ORL, Otorhinolaryngology; MS, multidisciplinary sciences; MRE, medicine, research, and experimental; BS, behavioral sciences; FST, food science and technology; NEU, neurosciences; PHYS, physiology; VIR, virology; BMB, biochemistry and molecular biology; CB, cell biology.

Figure 2 visually represents the co-citation relationships among the most influential journals in COVID-19 related OD research. The analysis highlights the dominance of medicine, general, and internal journals (e.g., *New England Journal of Medicine, Lancet*) in the red area, while the blue area is dominated by otorhinolaryngology journals (e.g., *European Archives of Oto-Rhino-Laryngology, International Forum of Allergy* & *Rhinology*). The yellow area is a cluster of neurology journals, including *Frontiers in Neurology*, and the green area is dominated by comprehensive journals such as *Nature* and *Science*. This illustration demonstrates a pronounced interdisciplinary cross-fertilization among COVID-19 related OD research themes, as evidenced by the co-citation relationships among journals in the red, blue, yellow, and green regions.

### 3.2. Most co-cited references

**Figure 3:**
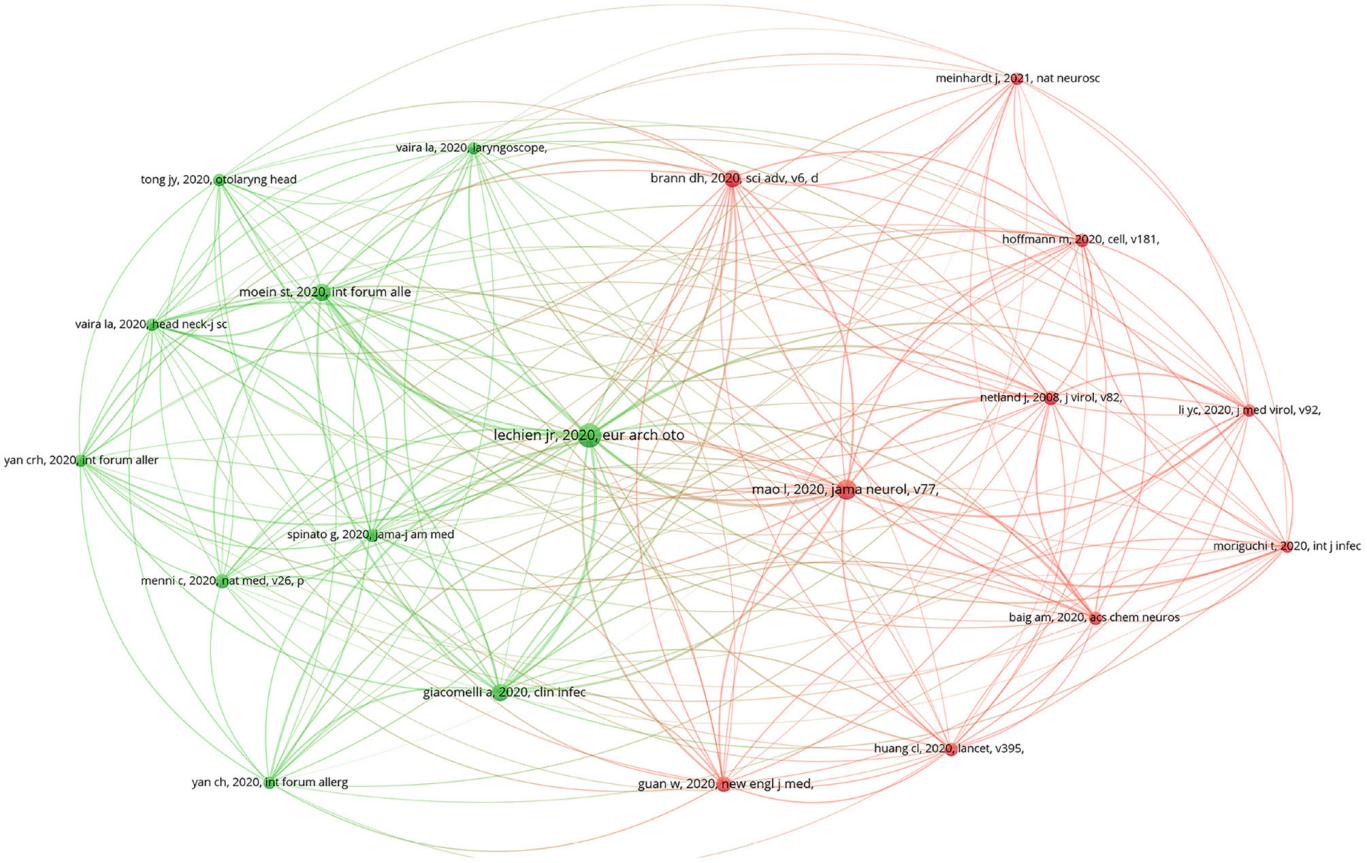
The mapping of cited sources co-citation analysis.

As depicted in Figure 3, the results of the co-citation analysis of highly cited references are presented, with the 20 most frequently cited references highlighted. The selection criteria of these references were established based on a minimum citation frequency of 128, out of a total of 40,340 cited references in the dataset. These highly cited references serve as indicators of the key topics and influential works in the field.

Figure 3 and Table 4 present the results of the highly cited reference analysis in the COVID-19 related OD research field. The selection criteria for these cited references were a citation frequency of over 128, and 20 references met this criterion among the 40,340 cited references in the dataset. The results are sorted by the TLS metric, and the LCS and Global Citation Score (GCS) are also listed.

**Table 4:** Highly total link strength cited references (Top 20).

**No**.	**Literature title**	**Type**	**Year**	**LCS**	**GCS**	**TLS**
1	Olfactory and gustatory dysfunctions as a clinical presentation of mild-to-moderate forms of the coronavirus disease (COVID- 19): a multicenter European study (Lechien et al., 2020).	Article	2020	517	2, 865	1, 989
2	Neurologic manifestations of hospitalized patients with coronavirus disease 2019 in Wuhan, China (Mao et al., 2020).	Article	2020	358	6, 641	1, 529
3	Self-reported olfactory and taste disorders in patients with severe acute respiratory coronavirus 2 infection: a cross-sectional study (Giacomelli et al., 2020).	Letter	2020	266	1, 519	1, 297
4	Smell dysfunction: a biomarker for COVID-19 (Moein et al., 2020).	Article	2020	256	781	1, 217
5	Non-neuronal expression of SARS-CoV-2 entry genes in the olfactory system suggests mechanisms underlying COVID-19- associated anosmia (Brann et al., 2020).	Article	2020	252	903	1, 057
6	Severe acute respiratory syndromecoronavirus infection causes neuronal death in the absence of encephalitis in mice transgenic for human ACE2 (Netland et al., 2008).	Article	2008	192	1, 358	904
7	Alterations in smell or taste in mildly Symptomatic outpatients with SARS- CoV-2 infection (Spinato et al., 2020).	Letter	2020	167	732	806
8	Objective evaluation of anosmia and ageusia in COVID-19 patients: single-center experience on 72 cases (Vaira et al., 2020a).	Article	2020	149	497	758
9	Clinical characteristics of coronavirus disease 2019 in China (Guan et al., 2020).	Article	2020	207	30, 205	749
10	Evidence of the COVID-19 virus targeting the CNS: tissue distribution, host-virus interaction, and proposed neurotropic mechanisms (Baig et al., 2020).	Editorial Material	2020	154	2, 395	746
11	Association of chemosensory dysfunction and COVID-19 in patients presenting with influenza-like symptoms (Yan C. H. et al., 2020).	Article	2020	133	864	702
12	Self-reported olfactory loss associates with outpatient clinical course in COVID-19 (Yan C. R. H. et al., 2020).	Article	2020	134	348	692
13	The neuroinvasive potential of SARS- CoV2 may play a role in the respiratory failure of COVID-19 patients (Li et al., 2020).	Review	2020	146	2562	664
14	Real-time tracking of self-reported symptoms to predict potential COVID-19 (Menni et al., 2020).	Article	2020	177	1, 236	640
15	SARS-CoV-2 cell entry depends on ACE2 and TMPRSS2 and is blocked by a clinically proven protease inhibitor (Hoffmann et al., 2020).	Article	2020	167	17, 297	635
16	Anosmia and ageusia: common findings in COVID-19 patients (Vaira et al., 2020b).	Article	2020	135	796	633
17	A first case of meningitis/encephalitis associated with SARS-Coronavirus-2 (Moriguchi et al., 2020).	Article	2020	128	2, 164	618
18	Clinical features of patients infected with 2019 novel coronavirus in Wuhan, China (Huang et al., 2020).	Article	2020	160	50, 435	532
19	The prevalence of olfactory and gustatory dysfunction in COVID-19 patients: a systematic review and meta- analysis (Tong et al., 2020).	Review	2020	134	578	518
20	Olfactory transmucosal SARS-CoV-2 invasion as a port of central nervous system entry in individuals with COVID-19 (Meinhardt et al., 2021).	Article	2021	131	979	342

The LCS metric measures the number of citations between locally retrieved collections of literature, and can reflect the degree of attention given to specific literature within a particular field, similar to peer evaluation metrics. It can be seen from the results in Table 4 that the majority of the most influential literature in COVID-19 related OD research is concentrated in 2020 and 2021, with the exception of one research literature from 2008. The types of literature mainly involve Articles, Letters, Reviews, and Editorial Material. Both specialized literature with low GCS indicators and clinical research literature with high GCS indicators are represented.

These findings emphasize the significance of recent developments in the COVID-19 related OD research field, as well as the importance of considering both specialized and clinical research literature in evaluating the impact of this research area.

Figure 3 and Table 4 highlight the divided knowledge base of COVID-19 related OD research, with two main clusters identified based on the top 20 highly cited papers as determined by the VOSviewer LCS metrics. These clusters center around COVID-19 clinical research and specialized OD research topics.

The first cluster, which focuses on COVID-19 clinical research, is represented by the red area in Figure 3 and includes literature such as Netland et al. (2008), Baig et al. (2020), Brann et al. (2020), Guan et al. (2020), Hoffmann et al. (2020), Huang et al. (2020), Li et al. (2020), Mao et al. (2020), Moriguchi et al. (2020), and Meinhardt et al. (2021), among others. These articles delve into various aspects of COVID-19, including neurological symptoms, OD, the susceptibility of neurons to SARS-CoV-2, clinical features, and treatment options. In particular, Mao et al. (2020) conducted a case study on 214 COVID-19 patients in Wuhan, China and found a significant proportion of neurological symptoms, including central nervous system manifestations, peripheral nervous system manifestations, and skeletal muscle damage manifestations. Brann et al. (2020) explored the connection between SARS-CoV-2 infection of non-neuronal cells and OD in COVID-19 patients, while Netland et al. (2008) found that neurons were highly susceptible targets of SARS-CoV.

The second cluster of COVID-19 related OD research, as depicted by the green area in Figure 3, encompasses literature that focuses on specialized OD topics. This cluster comprises literature numbered 1, 3, 4, 7, 8, 11, 12, 14, 16, and 19 in Table 4. Literature No.1, with the highest LCS and TLS indicators among the specialized OD literature, is an investigation carried out by Lechien et al. (2020) who surveyed 417 patients with COVID-19 across 12 European hospitals. They found that olfactory and gustatory dysfunction were clinical manifestations of mild to moderate cases of COVID-19. In addition, literature numbered 3, 8, 11, and 19, as demonstrated by Giacomelli et al. (2020), Tong et al. (2020), Vaira et al. (2020a), and Yan C. H. et al. (2020), respectively, show that taste or smell impairment are symptoms commonly observed in SARS-CoV-2-positive hospitalized patients. Furthermore, literature numbered 4, 12, 14, and 16, as reported by Menni et al. (2020), Moein et al. (2020), Vaira et al. (2020b), and Yan C. R. H. et al. (2020), suggest that olfactory impairment is a feature of neoconiosis and that olfactory testing may be useful for identifying patients in need of early treatment or isolation. Lastly, Spinato et al. (2020) in literature No.7 assessed the prevalence, intensity, and duration of olfactory or taste alterations.

### 3.3. Emerging themes from the literature

In the present study, a keyword co-occurrence analysis was conducted on a corpus of 1,592 documents related to OD in the context of COVID-19. The VOSviewer tool was utilized to visualize the relationships between author keywords within this research area. The resulting graph presents author keywords as nodes, with larger nodes indicating a greater frequency of occurrence of these keywords. The line segments connecting these nodes represent the interrelatedness between the keywords, with thicker lines indicating a stronger association and shorter lines reflecting a closer connection.

Before conducting analysis in VOSviewer, a systematic data cleaning and term merging is carried out. For instance, in regards to keywords related to COVID-19, various terms such as “coronavirus disease 2019,” “coronavirus disease 2019 (COVID-19),” “COVID-19,” and “COVID-19” are standardized as “COVID-19.” This process of term merging and data cleaning is crucial in maintaining consistency in terminology and enabling accurate analysis.

**Figure 4:**
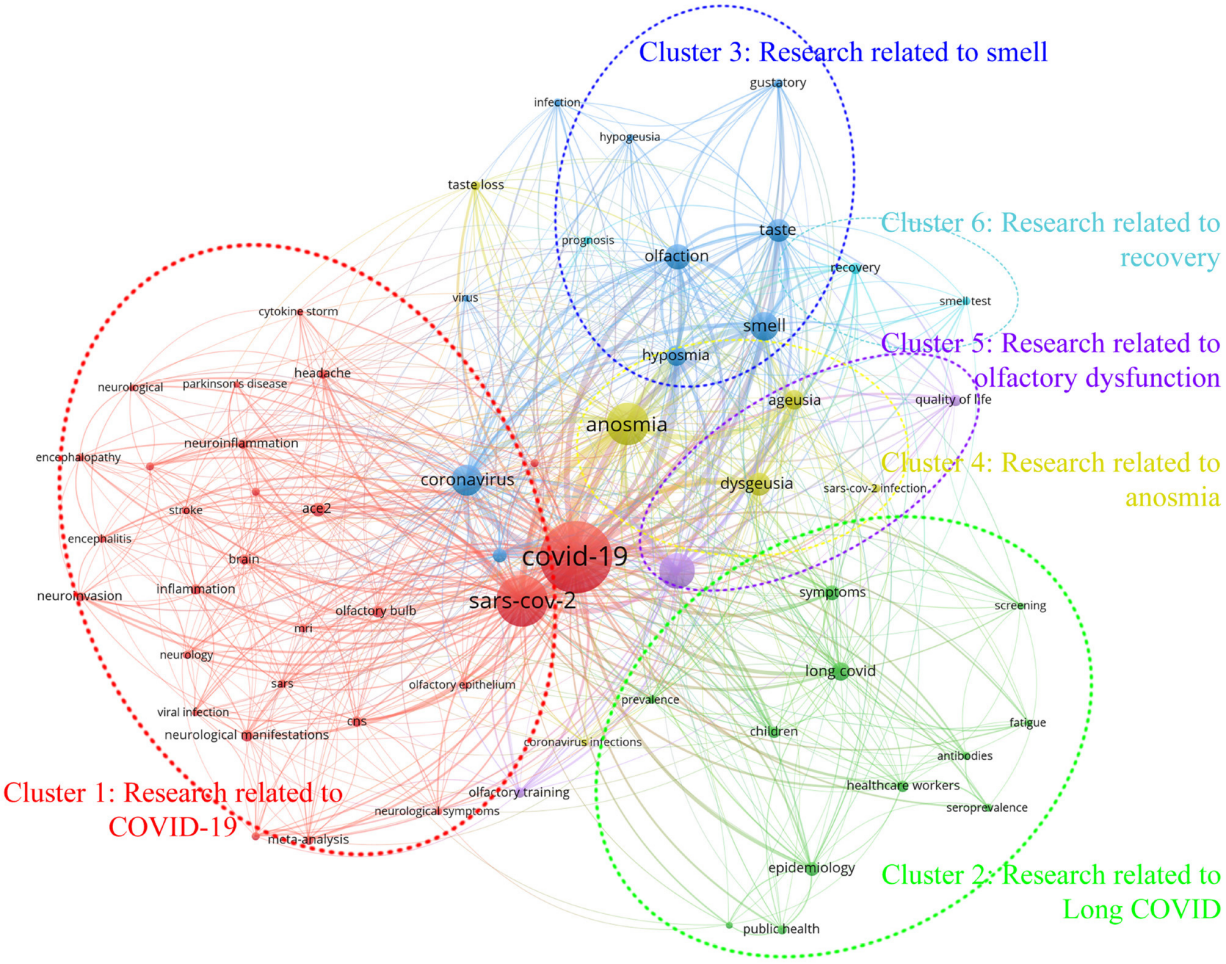
The mapping of author keywords co-occurrence analysis.

Subsequently, after carefully reviewing the dataset containing 2,649 author keywords, it was determined that those with a co-occurrence frequency exceeding 11 were deemed to be of particular significance. As a result, 62 keywords were selected and used to generate the keyword co-occurrence analysis graph depicted in Figure 4.

Figure 4 illustrates the results of the co-word analysis performed on the keywords utilized by authors in the field of COVID-19 and its related areas of study within the OD domain. The analysis reveals the existence of six clusters, each distinguished by a unique color, representing closely related hot topics. In order to succinctly convey the main concepts and hot topics of each cluster, Table 5 summarizes these findings.

**Table 5:** Research concepts and hot topics.

**Cluster**	**Concept**	**Nodes (*n*=62)**
1	COVID-19	ACE2, brain, CNS, COVID-19, cytokine storm, encephalitis, headache, encephalopathy, viral infection, inflammation, meta-analysis, MRI, neurodegeneration, neuroinflammation, neuroinvasion, neurological, neurological manifestations, neurological symptoms, neurology, neurotropism, olfactory bulb, olfactory epithelium, Parkinson's disease, risk factors, SARS, SARS-CoV-2, stroke, systematic review (*n* = 28)
2	Long COVID	Antibodies, children, epidemiology, fatigue, healthcare workers, infectious disease, long COVID, prevalence, public health, screening, seroprevalence, symptoms (*n* = 12)
3	Smell	Coronavirus, gustatory, hypogeusia, hyposmia, infection, olfaction, pandemic, smell, taste, virus (*n*=10)
4	Anosmia	Ageusia, anosmia, coronavirus infections, dysgeusia, SARS-CoV-2 infection, taste loss (*n* = 6)
5	Olfactory dysfunction	Olfactory dysfunction, olfactory training, quality of life (*n* = 3)
6	Recovery	Prognosis, recovery, smell test (*n* = 3)

The results of the high-frequency author keyword co-occurrence analysis, as depicted in Figure 4 and summarized in Table 5, demonstrate the major hotspots and emerging themes in COVID-19 related research within the OD domain. Utilizing the VOSviewer tool, the findings indicate that these themes can be broadly categorized into six clusters: COVID-19, long COVID, smell, anosmia, olfactory dysfunction, and recovery research. This information provides valuable insight into the current state and direction of COVID-19 related research.

Cluster 1 (Red): An examination of COVID-19 research was conducted and revealed the largest cluster, comprising 28 keywords. The most prevalent keyword, “COVID-19” (occurrences = 1,052, total link strength = 2,492), was found to be closely related to “SARS-CoV-2,” “ACE2,” “olfactory bulb,” “CNS,” “neurological manifestations,” and other relevant terms.

Cluster 2 (Green): The second significant cluster was identified as related to the phenomenon of long COVID, which consisted of 12 keywords. The most frequently occurring keyword, “long COVID” (occurrences = 70, total link strength = 154), was found to be associated with “symptoms,” “epidemiology,” “children,” “healthcare workers,” “prevalence,” and other related keywords.

Cluster 3 (Blue): This cluster, consisting of 10 keywords, was found to be related to the research area of smell. The most frequent keyword, “smell” (occurrences = 173, total link strength = 688), was shown to be one of many neurological symptoms affected by the “coronavirus” and “virus,” impacting sensory perception and resulting in related neurological symptoms such as “olfaction,” “taste,” “gustatory,” “hyposmia,” “hypogeusia,” and others.

Cluster 4 (Yellow): Our analysis revealed the emergence of a cluster centered around the theme of anosmia, with six keywords identified. The keyword “Anosmia” had the highest frequency of occurrence (occurrences = 370, total link strength = 1,259) and was found to be strongly associated with “dysgeusia,” “ageusia,” “taste loss,” and “coronavirus infections,” and “SARS-CoV-2 infection.”

Cluster 5 (Purple): Another cluster was identified that pertains to OD, encompassing three keywords. The keyword “olfactory dysfunction” was the most frequently occurring among these keywords (occurrences = 255, total link strength = 788) and was observed to have a causal relationship with “olfactory training” and “quality of life.”

Cluster 6 (Turquoise): The sixth cluster identified in our analysis pertained to the theme of Recovery, with three keywords identified. The keyword “recovery” was the most frequently occurring among these keywords (occurrences = 29, total link strength = 113) and was observed to be associated with “smell test” and “prognosis.”

The largest of the six study clusters discussed is COVID-19, where evidence has emerged that SARS-CoV-2 infecting Angiotensin-converting enzyme 2 (ACE2) leads to olfactory impairment in patients with neocoronary pneumonia (Bilinska et al., 2020). The presence of the virus in the olfactory epithelium and bulb further highlights the critical role that olfaction plays in the potential pathways of SARS-CoV-2 entry into the central nervous system (CNS) (Lima et al., 2020; Klingenstein et al., 2021). The growing body of research supports the notion that SARS-CoV-2 can target the nervous system, leading to various neurological manifestations, including loss of smell and taste (Divani et al., 2020). The second largest cluster is “long COVID,” which refers to symptoms, signs, and adverse reactions that persist for a prolonged period following neocoronavirus infection. Among the symptoms of Long COVID, Liao et al. (2022) have noted that olfactory and gustatory disturbances may be the primary symptoms in patients with long COVID-19. A significant proportion of patients reportedly develop persistent chemosensory impairments, including olfactory and taste disturbances, ranging from 3 months to 2 years after the onset of symptoms.

Studies from both the COVID-19 and long COVID clusters suggest that SARS-CoV-2 can result in neurological manifestations, including loss of smell, which may present as a persistent phenomenon in long COVID patients.

Within the six research clusters, four clusters, including smell, anosmia, olfactory dysfunction, and recovery, are focused on OD and its related themes. Multiple studies have reported that the senses of smell and taste are the most frequently impacted in patients with neocoronary pneumonia, with OD and taste impairment being key symptoms of the illness (Lechien et al., 2020; Yan C. H. et al., 2020; Ferrulli et al., 2022). Among them, OD is classified into two levels: olfactory loss (anosmia) and olfactory decline (hyposmia). The accurate detection, diagnosis, treatment, olfactory training, recovery, and prognosis of OD in neocoronary pneumonia patients are essential to enhance their quality of life.

## 4. Discussion

### 4.1. Research trend under the perspective of time

**Figure 5:**
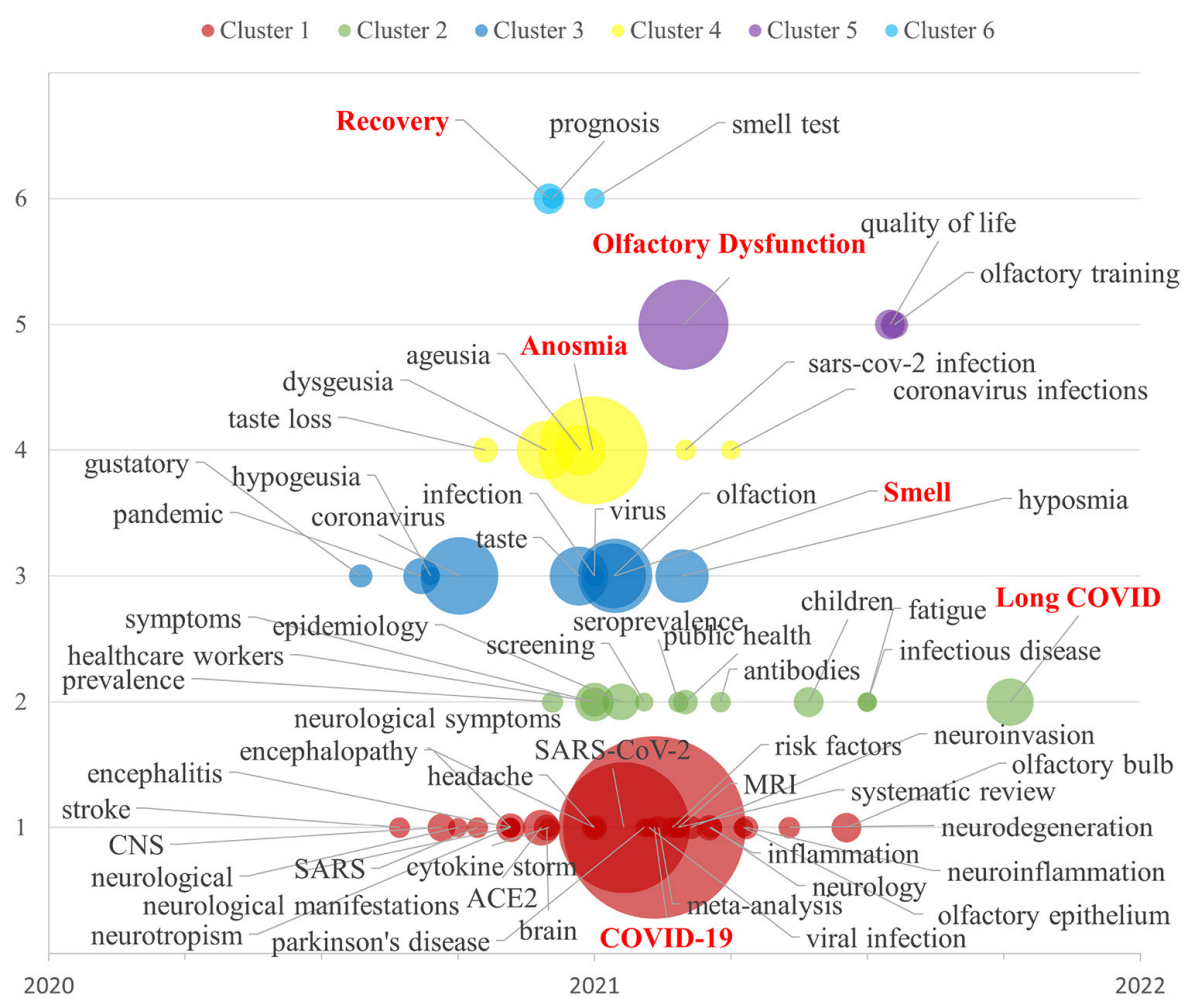
The mapping of keywords temporal evolution.

Given the rapidly evolving nature of COVID-19, with its alarming rate of transmission and the emergence of new mutant strains, it is imperative to have a comprehensive understanding of the spatiotemporal evolution of related research results. Therefore, in this paper, based on the clusters of Figure 4 and their keywords, and subsequently combined with the average year of publication values of each keyword counted in VOSviewer, we produced the mapping of keywords in six clusters of COVID-19 related OD temporal evolution, as shown in Figure 5, thus revealing the temporal trends of related studies.

As depicted in Figure 5, the temporal evolution of COVID-19 related OD research can be characterized by three distinct phases. The second phase, which began in the beginning of 2021, is the phase where the majority of the keywords in the six clusters of COVID-19 related OD research show a concentrated peak, particularly for keywords such as COVID-19, smell, anosmia, olfactory dysfunction, and recovery. This suggests that the world was facing its most challenging moment in terms of COVID-19 during this phase, with a significant shortage of medical resources in several countries and an immediate need for extensive COVID-19-related research efforts. The first phase encompasses the period prior to the start of 2021, during which the impact of COVID-19 on patients' sense of taste and smell was gradually being recognized. This phase saw the initial attention from researchers toward the topics of SARS, CNS, ACE2, and taste loss. Finally, the third phase, which began in 2021, has seen a consolidation of the objective fact that COVID-19 causes OD symptoms. This stage has witnessed a shift in research focus toward new areas, such as long COVID, olfactory bulb, fatigue, olfactory training, quality of life, among others.

### 4.2. Suggestions on future research

#### 4.2.1. Understanding the mechanisms of OD

Several studies have explored the mechanisms underlying OD in COVID-19 patients. Pujadas et al. (2021) conducted a molecular profiling autopsy study and revealed the damage to the olfactory bulb caused by the New Coronary Pneumonia. Sharma et al. (2021) reviewed the entry pathways and pathogenic mechanisms of SARS-CoV-2 into the central nervous system, including the olfactory and retinal nervous systems. Najafloo et al. (2021) examined the mechanisms associated with olfactory impairment in neocoronary pneumonia, including olfactory cleft syndrome, local inflammation, apoptosis of olfactory cells, and damage to olfactory neurons and stem cells.

Sodagar et al. (2022) reviewed the pathological features, neuroinflammatory mechanisms, and potential treatments of SARS-CoV-2 in the brain, finding strong infection of the olfactory bulb, thalamus, and brainstem. Karimian et al. (2022) explored the molecular mechanism of SARS-CoV-2-induced olfactory deficit, suggesting that OD may be a temporary or long-term complication caused by olfactory neuroepithelial disorders, with the Delta and Omicron strains relying on TMPRSS2 to enter cells and inducing inflammation, apoptosis, and neuronal damage.

Despite these advances, there remains a significant knowledge gap regarding the possible mechanisms leading to olfactory loss (Dunai et al., 2022). Further studies are urgently needed to better understand and identify the causes of OD in COVID-19 patients, in order to improve prevention and treatment strategies (de Melo et al., 2021).

#### 4.2.2. Assessment of OD

In recent years, various tools and methods have been employed to evaluate OD in patients with COVID-19. For instance, Duff et al. (2022) utilized brain magnetic resonance imaging (MRI) to assess the neuropsychiatric consequences of neocoronary pneumonia, demonstrating the high reliability of multiple imaging derived phenotypes (IDPs) in measuring the impact of SARS-CoV-2 infection on the brain through potential pathogenic mechanisms. Melkumyan et al. (2022) conducted a cross-sectional study that analyzed the sensitivity to olfaction and trigeminal nerve-triggered olfaction, as well as the ability to distinguish between different odors, in patients recovering from neocoronary pneumonia and revealed pathological changes in olfactory and trigeminal perceptual abilities that result in OD. Kim and Min (2022) proposed a psychophysical assessment system for OD in neocoronary pneumonia patients using a universal odorant that is free from the risk of viral transmission, which may provide early diagnosis and management of such patients. Ciofalo et al. (2022) used the Visual Analogue Scale (VAS) to prospectively evaluate nasal and olfactory symptoms in 162 neocoronary pneumonia patients. Gupta et al. (2022) utilized the Novel Anosmia Screening at Leisure (NASAL) patient report to assess olfactory perception, demonstrating its efficacy in identifying OD in patients.

These studies suggest that the use of OD assessment tools or methods can aid in our understanding of the prevalence of the disease and facilitate the development of effective treatments. However, further research is necessary in this field, particularly to establish standardized assessments that can more accurately measure OD in patients with COVID-19.

#### 4.2.3. Interventions of OD

In addressing OD in patients with neocoronary pneumonia, a range of therapeutic approaches have been evaluated, including oral supplementation, topical medications, nasal rinses, and olfactory training.

Hosseinpoor et al. (2022) and Vaira et al. (2022) studied the effect of intranasal corticosteroid treatment on long-term OD recovery caused by COVID-19. At the same time, Veronese et al. (2022) found that the combination of olfactory rehabilitation and oral supplementation of palmitoyl ethanolamine and lidocaine can improve the treatment effect of post-conjunctivitis OD. A review by Gao et al. (2022) reported that topical herbal therapies demonstrated positive effects in the treatment of OD. Additionally, a study by Forouzanfar et al. (2022) revealed that diets containing pomegranate juice and lacquer sap were effective in reducing symptoms such as olfactory and gustatory dysfunction in patients with neoconjunctivitis.

As for olfactory training, Hwang et al. (2023) evaluated the impact of olfactory training on OD in patients with neocoronary pneumonia. It is found that olfactory training was effective in improving OD caused by neocoronary pneumonia both in the acute and chronic phases. Khan et al. (2022) conducted a randomized clinical trial on the efficacy of combined visual-olfactory training in patients with olfactory loss due to neocoronary pneumonia. The results of the trial suggest that bimodal visual-olfactory training may benefit patients with neocoronary pneumonia, although the efficacy of fragrance treatment has not yet been established.

In conclusion, multiple drug and non drug interventions have shown good results in the OD treatment of patients with COVID-19. Further research is needed to determine the most effective treatment and explore the potential benefits of joint intervention. In addition, personalized intervention is needed for different groups (such as children, the elderly and patients with potential health conditions) to better understand the impact of OD on these groups and develop personalized intervention measures.

#### 4.2.4. Long COVID prognosis of OD

Several studies have investigated the prognosis of OD in patients with neocoronary pneumonia. Mendonca et al. (2022) reported that OD is more prevalent in patients with neocoronary pneumonia than in those with severe disease, and that the presence of olfactory hyposmia/anosmia may indicate a favorable prognosis in neocoronary pneumonia. Tan et al. (2022) found that a significant proportion of patients with neocoronary pneumonia may experience long-lasting changes in their sense of smell or taste, potentially exacerbating the impact of long COVID.

Ho et al. (2022) conducted autopsy assessments on patients with neocoronary pneumonia and found that the infection is associated with axonal damage and microangiopathy in the olfactory tissues, resulting in severe and permanent OD. A review by Ibrahim et al. (2022) explored the potential determinants of poor prognosis for neurological symptoms in neocoronary pneumonia and reported that the olfactory nerve is the most commonly affected cranial nerve, resulting in olfactory loss.

In conclusion, the long-term prognosis of OD in patients with neocoronary pneumonia is still hard to predict. Further studies are needed to ensure its recovery rate and persistence. This will be important in providing guidance and developing treatments for patients with persistent psychosis.

## 5. Conclusion

The COVID-19 pandemic has been a catastrophic public health event, with infection contributing to a significant increase in the global prevalence of OD symptoms, affecting the quality of life of long-term COVID-19 patients and posing a major challenge to human health. To address this challenge, it is crucial to raise awareness and further explore the underlying mechanisms of OD through research. Our study analyzed 1,592 publications related to COVID-19 related OD in the Web of Science database, demonstrating the broad interest of researchers from different disciplines and countries. The most influential findings in the field were published in otorhinolaryngology, medicine, general and internal medicine, virology, and neuroscience journals. The study identified six research hotspots, including COVID-19, long COVID, smell, anosmia, recovery, and olfactory dysfunction. These findings provide valuable insight into the temporal trends in COVID-19 related OD research and will aid future researchers in understanding and developing effective assessments, interventions, and prognostic options. Ultimately, a better understanding of the underlying mechanisms of OD in COVID-19 patients will be crucial in addressing the lasting legacy of the pandemic on human health.

## Author contributions

YM and WB contributed to the writing and conception of the professional part of the study. ZY and JT contributed to the implementation of bibliometric methods and mapping. YM, ZY, and JT drafted the manuscript and performed the data analysis. WB and JT revised the manuscript and made critical suggestions on this work. All authors contributed to the article and approved the submitted version.
